# Aortic Annular Sizing Using Novel Software in Three-Dimensional Transesophageal Echocardiography for Transcatheter Aortic Valve Replacement: A Systematic Review and Meta-Analysis

**DOI:** 10.3390/diagnostics11050751

**Published:** 2021-04-22

**Authors:** Chanrith Mork, Minjie Wei, Weixi Jiang, Jianli Ren, Haitao Ran

**Affiliations:** 1 Department of Ultrasound, The Second Affiliated Hospital of Chongqing Medical University, Chongqing 400010, China; chanrithmork@gmail.com (C.M.); minjiewei1996@163.com (M.W.); doctorxipishi@163.com (W.J.); 13883925580@163.com (J.R.); 2 Chongqing Key Laboratory of Ultrasound Molecular Imaging, Chongqing 400010, China

**Keywords:** novel software, three-dimensional transesophageal echocardiography, multidetector computed tomography, transcatheter aortic valve replacement

## Abstract

(1) Background: We performed this study to evaluate the agreement between novel automated software of three-dimensional transesophageal echocardiography (3D-TEE) and multidetector computed tomography (MDCT) for aortic annular measurements of preprocedural transcatheter aortic valve replacement (TAVR); (2) Methods: PubMed, EMBASE, Web of Science, and Cochrane Library (Wiley) databases were systematically searched for studies that compared 3D-TEE and MDCT as the reference standard for aortic annular measurement of the following parameters: annular area, annular perimeter, area derived-diameter, perimeter derived-diameter, maximum and minimum diameter. Meta-analytic methods were utilized to determine the pooled correlations and mean differences between 3D-TEE and MDCT. Heterogeneity and publication bias were also assessed. Meta-regression analyses were performed based on the potential factors affecting the correlation of aortic annular area; (3) Results: A total of 889 patients from 10 studies were included in the meta-analysis. Pooled correlation coefficients between 3D-TEE and MDCT of annulus area, perimeter, area derived-diameter, perimeter derived-diameter, maximum and minimum diameter measurements were strong 0.89 (95% CI: 0.84–0.92), 0.88 (95% CI: 0.83–0.92), 0.87 (95% CI: 0.77–0.93), 0.87 (95% CI: 0.77–0.93), 0.79 (95% CI: 0.64–0.87), and 0.75 (95% CI: 0.61–0.84) (Overall *p* < 0.0001), respectively. Pooled mean differences between 3D-TEE and MDCT of annulus area, perimeter, area derived-diameter, perimeter derived-diameter, maximum and minimum diameter measurements were −20.01 mm^2^ ((95% CI: −35.37 to −0.64), *p* = 0.011), −2.31 mm ((95% CI: −3.31 to −1.31), *p* < 0.0001), −0.22 mm ((95% CI: −0.73 to 0.29), *p* = 0.40), −0.47 mm ((95% CI: −1.06 to 0.12), *p* = 0.12), −1.36 mm ((95% CI: −2.43 to −0.30), *p* = 0.012), and 0.31 mm ((95% CI: −0.15 to 0.77), *p* = 0.18), respectively. There were no statistically significant associations with the baseline patient characteristics of sex, age, left ventricular ejection fraction, mean transaortic gradient, and aortic valve area to the correlation between 3D-TEE and MDCT for aortic annular area sizing; (4) Conclusions: The present study implies that 3D-TEE using novel software tools, automatically analysis, is feasible to MDCT for annulus sizing in clinical practice.

## 1. Introduction

Transcatheter aortic valve replacement (TAVR) has evolved as a safe and effective intervention alternative to surgical aortic valve replacement (SAVR), allowing high-risk, intermediate-risk, and recently low-surgical risk patients to be treated for severe symptomatic aortic stenosis (AS) by replacing the native valve with a bioprosthetic valve [1,2,3,4,5,6,7].

Accurate sizing of the aortic annulus is a crucial step towards the success of TAVR and is the preferred source of the transcatheter heart valve (THV) selection [8,9,10,11]. However, paravalvular aortic regurgitation (PAR), device embolization, aortic root rupture, conduction disturbances, and prosthesis-patient mismatch (PPM) have all been linked to incorrect valve selection [12,13,14,15].

A multidetector computed tomography (MDCT) procedure for pre-TAVR planning has been recommended to be the gold standard method due to the ability to accurately measure the dimensions of the ascending aorta, the aortic root, and the aortic annulus (AA) [16,17,18,19]. However, renal impairment increases the risk of contrast-induced nephropathy, making MDCT unavailable for patients with compromised renal function [20,21,22]. As a result, three-dimensional transesophageal echocardiography (3D-TEE), which does not require iodinated contrast, may be a useful imaging tool during TAVR, providing accurate measurements of the aortic root and geometry as an alternative to MDCT [23,24,25,26,27].

Recently, many studies performing automated quantification software in 3D-TEE data provided a high agreement compared to the MDCT, and reduced time consuming with less observer-dependent compared to 3D-TEE manual technique or direct planimetry for aortic annular measurements [28,29,30,31].

Therefore, the purpose of this systematic review and meta-analysis was to evaluate evidence on the comparison between 3D-TEE automated software and MDCT for TAVR annular measurements.

## 2. Materials and Methods

This systematic review was conducted in agreement with the PRISMA (Preferred Reporting Items for Systematic Reviews and Meta-Analyses) statement [32]. The PRISMA checklist was used to describe our study lists in Table S1.

### 2.1. Search Strategy

A systematic search of the PubMed, EMBASE, Web of Science, and Cochrane Library (Wiley) databases was performed by two reviewers (C.M., M.W.) independently to identify relevant studies published till 30 August 2020. Supplementary Materials lists the searched terms in Table S2.

### 2.2. Eligibility Criteria

Studies were considered eligible in the meta-analysis if they met the following criteria: (1) studies which included patients with symptomatic aortic stenosis or/and underwent TAVR for aortic valve replacement; (2) studies evaluating the aortic annulus measurements by automated or semiautomated 3D-TEE as an index test and MDCT as a reference standard; (3) studies which assessed the correlation coefficient or agreement between the finding of MDCT and 3D-TEE for aortic annular measurements; and (4 ) original published articles as type of study.

Exclusion criteria were (1) single-arm studies; (2) studies using 3D-TEE manually as the measurement technique; (3) studies with patients presenting normal aortic valve; (4) non-English studies; and (5) case studies.

### 2.3. Data Extraction

Data were independently extracted by two investigators (C.M., W.J.). We extracted the following information from published papers (1) study characteristics (author, year of publication, total number of patients, study design, study period, hospital or institution, and country; (2) patient characteristics (sex, age, body mass index, body surface area, atrial fibrillation, hypertension, diabetes mellitus, aortic valve area, mean transaortic gradient, and left ventricular ejection fraction; (3) imaging characteristics (3D-TEE technique and vendor, MDCT technique and vendor, measurement phase, software used for 3D-TEE annulus sizing); (4) study outcomes as assessed in the individual studies(correlation coefficient and sample size for pooling correlation; mean, standard deviation, and sample size for pooling mean difference). There were six parameters used for comparison in annular sizing between 3D-TEE and MDCT: annular area, annular perimeter, area derived-diameter, perimeter derived-diameter, maximum and minimum diameter.

### 2.4. Quality Assessment

We evaluated the methodological quality of individual studies using the QUADAS−2 tool and summarized quality assessments per fulfilled QUADAS-2 domains: patient selection, index test, reference standard, and flow and timing. Each domain was assessed in terms of risk of bias and rated as “low”, “high” or “unclear”. Concerns regarding applicability were assessed only for the first three domains and categorized into “low”, “high”, or “unclear”. Signaling questions could be answered with “yes”, “no”, or “unclear” [33].

### 2.5. Data Synthesis and Analyses

The correlations between novel software 3D-TEE and MDCT measurements from each study were transformed to Fisher z correlation coefficients for analysis and backtransformed to report as correlation coefficient (r) and 95% CIs, while the mean differences were pooled and reported as mean difference and 95% CIs [34]. Random-effects models of DerSimonian and Laird were used to calculate pooled effect sizes. The Cochran Q statistic and the I2 test were used to assess studies heterogeneity [35]. Funnel plot and Egger’s regression test were used to assess for potential publication bias [36].

The influencing factors of sex, age, left ventricular ejection fraction, aortic valve area, and mean transaortic gradient were analyzed using a meta-regression analysis (The DerSimonian–Laird method) on the correlation of aortic annular area.

Statistical analyses were performed using the “meta” package in R software (Version 4.0.3, R Foundation for Statistical Computing).

## 3. Results

### 3.1. Study Characteristics

**Figure 1:**
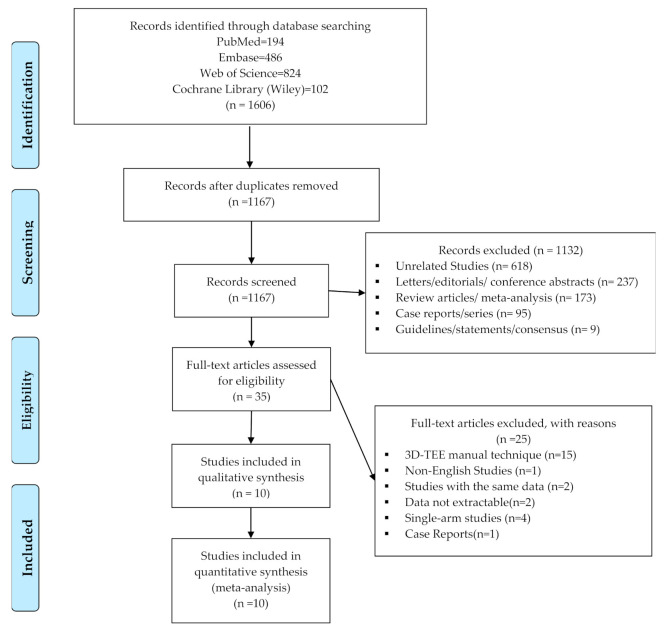
PRISMA flow diagram of the study selection process. 3D-TEE = Three-dimensional transesophageal echocardiography, n = Number of literatures, PRISMA = Preferred Reporting Items for Systematic Reviews and Meta-analyses.

The article selection process is described in detail in Figure 1. After the study selection process, a total of 889 patients from 10 studies were included in the meta-analysis [37,38,39,40,41,42,43,44,45,46]. The detailed characteristics of the included studies are reported in Table 1, Table 2 and Table 3. The sizes of the original study populations ranged from 31 to 175 patients, with the mean patient ages ranging from 80 to 88 years. All individual studies were retrospective study. Six studies included were reported using the automated techniques [38,40,41,42,45,46], while the other four studies reported semiautomated techniques [37,39,43,44] as the using novel software of 3D-TEE comparing to MDCT for annular measurements to calculate weighted effect size of this study.

### 3.2. Quality Assessment

**Figure 2:**
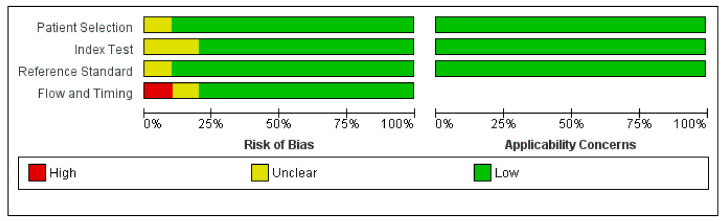
Quality assessment of included studies. Risk of bias and applicability of concerns domains are presented as percentages based on modified Quality Assessment of Diagnostic Accuracy Studies-2 tool. Each bar shows percentage of studies with high (red), unclear (yellow), and low (green) risks of bias and applicability of concerns.

The results of the QUADAS-2 assessment are presented in Figure 2. Overall, one study (10%) showed a high risk of bias in the “flow” and “timing” domains. Concerns regarding applicability were rated “low” in all the domains.

### 3.3. Correlation between 3D-TEE and MDCT for Annulus Measurements

**Figure 3:**
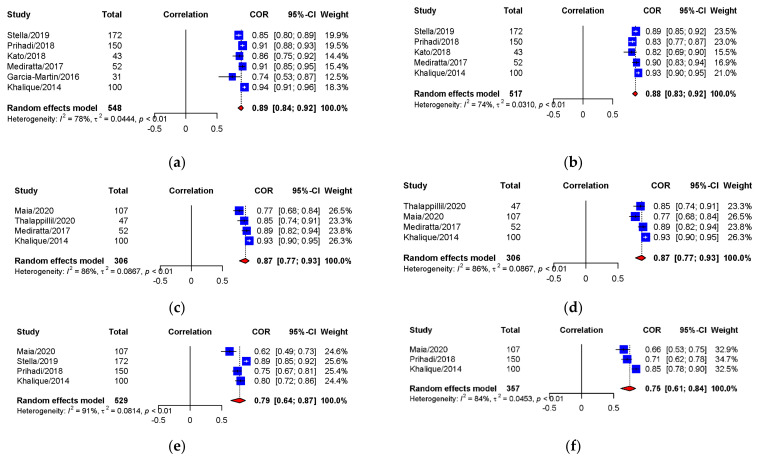
Forest plots of correlation between 3D-TEE and MDCT for annular sizing parameters. (**a**) Aortic annular area; (**b**) Aortic annular perimeter; (**c**) Annular area-derived diameter; (**d**) Annular perimeter-derived diameter; (**e**) Annular maximum diameter; (**f**) Annular minimum diameter. 3D-TEE = Three-dimensional transesophageal echocardiography, MDCT = Multidetector computed tomography, CI = Confidence interval, COR = Correlation coefficient.

**Figure 4:**
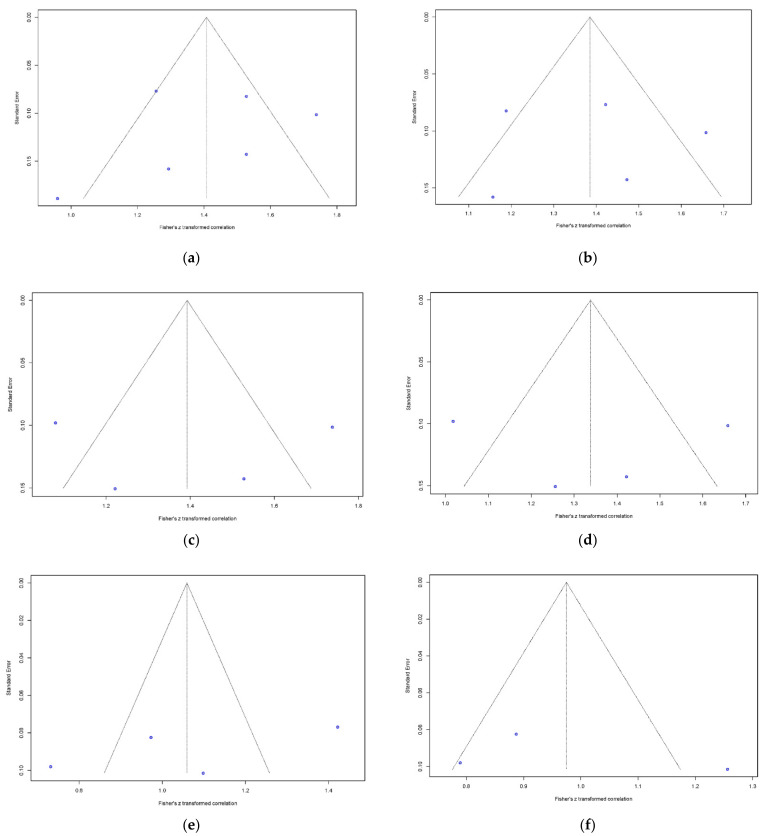
Funnel plots to detect publication bias of correlation between 3D-TEE and MDCT. Bias and *p* values of asymmetry derived from Egger’s test are shown for annular sizing parameters. (**a**) Aortic annular area (bias = −1.40, *p* = 0.67); (**b**) Aortic annular perimeter (bias = 0.15, *p* = 0.97); (**c**) Annular area-derived diameter (bias = 0.11, *p* = 0.99); (**d**) Annular perimeter-derived diameter (bias = 0.92, *p* = 0.92); (**e**) Annular maximum diameter (bias = −16.67, *p* = 0.34); (**f**) Annular minimum diameter (bias = 10.36, *p* = 0.69). 3D-TEE = Three-dimensional transesophageal echocardiography, MDCT = Multidetector computed tomography.

The pooled correlation coefficients between 3D TEE and MDCT of annulus area, perimeter, area derived-diameter, perimeter derived-diameter, maximum and minimum diameter measurements were strong 0.89 (95% CI: 0.84–0.92), 0.88 (95% CI: 0.83–0.92), 0.87 (95% CI: 0.77–0.93), 0.87 (95% CI: 0.77–0.93), 0.79 (95% CI: 0.64–0.87), and 0.75 (95% CI: 0.61–0.84) (Overall *p* < 0.0001), respectively. Figure 3 summarizes the forest plots with provided heterogeneity test results. Figure 4 presents the funnel plots of each parameter showed relatively symmetric funnel plots without significant publication bias (*p* > 0.05).

### 3.4. Mean Difference between 3D-TEE and MDCT for Annulus Measurements

**Figure 5:**
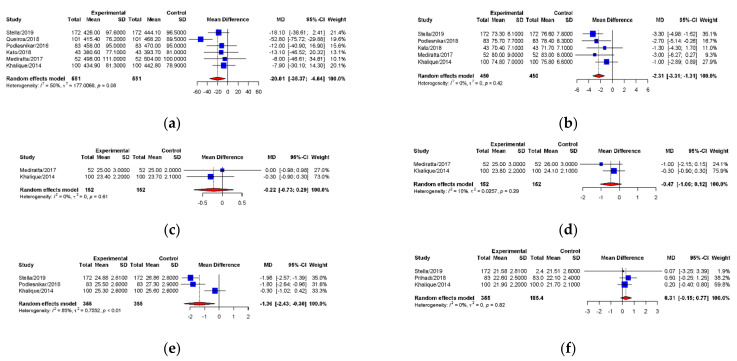
Forest plots of mean differences between 3D-TEE and MDCT for annular sizing parameters. *p* values derived from the pooled mean difference of each parameters are shown. (**a**) Aortic annular area (*p* = 0.011); (**b**) Aortic annular perimeter (*p* < 0.0001); (**c**) Annular area-derived diameter (*p* = 0.40); (**d**) Annular perimeter-derived diameter (*p* = 0.12); (**e**) Annular maximum diameter (*p* = 0.012); (**f**) Annular minimum diameter (*p* = 0.18). 3D-TEE = Three-dimensional transesophageal echocardiography (Experimental), MDCT = Multidetector computed tomography (Control), CI = Confidence interval, MD = Mean difference, SD = Standard deviation.

**Figure 6:**
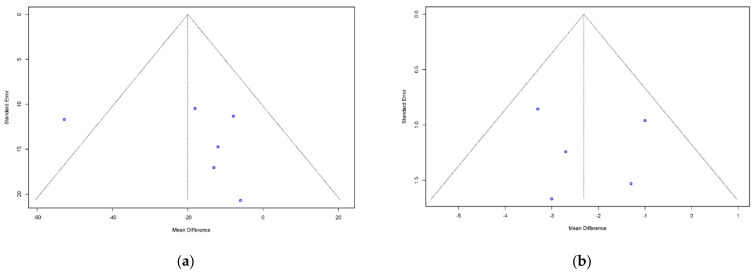

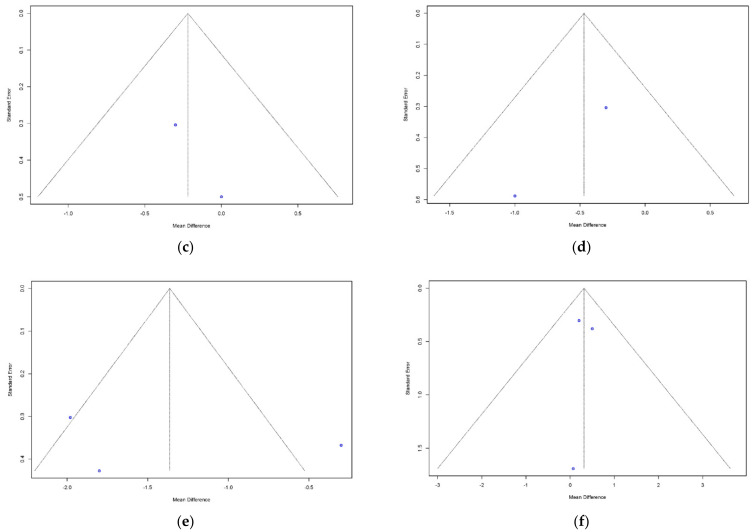
Funnel plots to detect publication bias of mean difference between 3D-TEE and MDCT. Bias and *p* values of asymmetry derived from Egger’s test are shown for annular sizing parameters. (**a**) Aortic annular area (bias = 1.72, *p* = 0.56); (**b**) Aortic annular perimeter (bias = 0.45, *p* = 0.84); (**c**) Annular area-derived diameter (Unable to test); (**d**) Annular perimeter-derived diameter (Unable to test); (**e**) Annular maximum diameter (bias = 4.62, *p* = 0.80); (**f**) Annular minimum diameter (bias = −0.03, *p* = 0.98). 3D-TEE = Three-dimensional transesophageal echocardiography, MDCT = Multidetector computed tomography.

The pooled mean differences between 3D-TEE and MDCT of annulus area, perimeter, area derived-diameter, perimeter derived-diameter, maximum and minimum diameter measurements were −20.01 mm2 ((95% CI: −35.37 to −0.64), *p* = 0.011), −2.31 mm ((95% CI: −3.31 to −1.31), *p* < 0.0001), −0.22 mm ((95% CI: −0.73 to 0.29), *p* = 0.40), −0.47 mm ((95% CI: −1.06 to 0.12), *p* = 0.12), −1.36 mm ((95% CI: −2.43 to −0.30), *p* = 0.012), and 0.31 mm ((95% CI: −0.15 to 0.77), *p* = 0.18), respectively. Figure 5 summarizes the forest plots with provided heterogeneity test results. Figure 6 presents the funnel plots of annulus area, perimeter, area derived-diameter, maximum and minimum diameter showed relatively symmetric funnel plots without significant publication bias (*p* > 0.05). However, annular area derived-diameter and annular perimeter derived-diameter could not be analyzed by Egger’s test because of only two studies reported.

### 3.5. Meta-Regression Analysis

There were no statistically significant (*p* > 0.05) associated to the baseline patient characteristics of sex, age, left ventricular ejection fraction, aortic valve area, and mean transaortic gradient to the correlation between 3D-TEE and MDCT annular area. The correlation between 3D-TEE and MDCT annular area of baseline patient characteristics presented in Table 4.

## 4. Discussion

The aortic root, which runs from the aortic valvular cusps’ basal attachment to the sinotubular junction, is a direct continuation of the left ventricular outflow tract. The aortic annulus diameter, the virtual ring, and the complex structure initially assumed to have a circular structure, is defined as the plane transecting the three aortic cusps’ lowest hinge -points, measured in systole to determine prosthesis size and prosthetic valve selection of the TAVR procedures [10,18,47,48].

In the TAVR prep-procedural planning, accurate measurement of the aortic annulus is critical of the successful protocol [11,18,19,27]. PAR, device embolization, aortic root rupture, conduction disturbances, coronary obstruction, and PPM have all been linked to the oversizing and the undersizing of THV valve selection [12,13,14,49]. The asymmetric shape of two-dimensional echocardiography (2D) made it ineffective for annular measurements, the disadvantage of providing a diameter measurement in a single plane and assuming a circular orifice that underestimated the cross-sectional measurements of 3D-based sizing with annulus’ elliptical geometry [9,37,38].

There has been significant interest in defining the shape by alternate imaging methods. Three modality imaging, 3D-TEE, cardiovascular magnetic resonance (CMR), and MDCT have emerged as the preferred, accurate, and reproducible techniques for cross-sectional dimensions, especially the annular area and perimeter [10,50,51,52,53]. Moreover, CMR may have a developing role for annular sizing, particularly in patients with renal dysfunction, though this is less implemented in the clinical work station [54]. Recently, MDCT is routinely used as the reference standard imaging modality in assessment of the aortic annulus for pre-TAVR procedure. However, MDCT has its own radiation exposure limits, higher costs, and contraindications to kidney failure and contrast allergy that are not appropriate for screening or repetitive control, particularly with the expansion of younger individual patients at lower surgical risk [7,20,21,55].

Notably, previous studies demonstrated that cross-sectional 3D-TEE manual measurements of the aortic annulus were underestimated obtained by MDCT, thus potentially resulting in prosthesis undersizing when implanted in the sizing algorithms recommended by manufactures [56,57,58,59]. When compared to the 3D-TEE manual technique, the introduction of novel software that utilized 3D-TEE data semiautomatically and automatically for the aortic annulus has dramatically used and allowed a more systematic approach to minimize the observer’s influence and time-consuming [40,43,44,45]. Additionally, the software tools demonstrated good to excellent agreement between 3D-TEE and MDCT for the measurements of aortic annulus with low interobserver and intraobserver variability [38,39,40,42].

Our meta-analysis is the first study that extracted data from 3D-TEE using novel automated software semiautomatically and automatically compare to MDCT for annular measurements. We found that 3D-TEE sizing for annular area, perimeter, area derived-diameter, perimeter derived-diameter, maximum and minimum diameter was strongly correlated with MDCT annulus measurements. However, the mean differences between 3D-TEE and MDCT of annular area, perimeter, area derived-diameter, perimeter derived-diameter, and maximum diameter were underestimated except the annular minimum diameter found to be slightly overestimated.

To the best of the authors’ knowledge, the two previous studies have attempted to methodically compare 3D-TEE and MDCT for TAVR sizing in the same patient population [60,61]. Both studies, however, basically used the 3D-TEE manual technique for annular measurements. Elkaryoni et al. reported a strong correlation for annular area (r = 0.84, *p* < 0.001), mean perimeter (r = 0.85, *p* < 0.001), and mean diameter (r = 0.80, *p* < 0.001), while the Bland–Altman plots showed the mean bias was −2.22 mm2 (95%CI, LOA: −12.79 to 8.36) for annular area between 3D-TEE manual and MDCT [60]. Rong et al. reported the strong correlations between 3D-TEE manual and MDCT for annular area (0.86 (95% CI, 0.80–0.90)), annular perimeter (0.89 (95%CI, 0.82–0.93)), and mean annular diameter (0.80 (95%CI, 0.70–0.87)) measurements, while the mean differences were slightly underestimated for annular area (−0.12 cm2 (95%CI, −0.24 to 0.00), *p* = 0.05), annular perimeter (−0.02 cm (95%CI, −0.65 to 0.61), *p* = 0.95), and mean annular diameter (−0.03 cm (95%CI, −0.15 to 0.10), *p* = 0.68) between 3D-TEE manual and MDCT measurements [61].

PAR is one of the most common complications seen after TAVR as a result of incorrect prosthesis size selection, and accurate aortic annulus measurements are crucial in reducing the frequency [1,37,59]. There has been investigation in studies that showed that the incidence of more than mild PAR was significantly lower when sizing of the aortic annulus was performed using MDCT compared with sizing performed by 2D echocardiography [52,62]. However, Khalique et al. utilized semiautomated 3D-TEE yielded the prediction of mild or greater PAR with equivalent accuracy to MDCT [37].

The novel software’s clinical impact on the selection prosthetic heart valve may have been shown with promising evidence mainly focused on the annular area and perimeter based−3D sizing. Kato et al., using the automated software “eSieVaves”, found that the 3D-TEE automated and semiautomated measurements were 72% and 78%, respectively, for the agreement to the size of prosthetic valve implanted [40]. Podlesnikar et al., using the automated software”4D Auto AVQ”, found that the agreement between 3D-TEE automated and MDCT measurements was an excellent agreement (kappa = 0.926) for selecting the same TAVR prosthesis size [41]. Prihadi et al., using the automated software “Aortic Valve Navigator (AVN)”, found that 3D-TEE automated and MDCT measurements yielded excellent agreement (kappa = 0.90) in prosthesis size choice in the majority of patients (93.3%) [42]. Queiros et al., using the automated software “Speqle 3D”, found that the measurements of 3D-TEE were good sizing agreement against the true implanted sizes (>77%) and against MDCT-based sizes (>88%) [43].

In the era of modern medicine, artificial intelligence has been the main subject to the revolution of cardiovascular multimodality imaging that recently also applied in echocardiography [63,64,65,66]. AI has the capacity to transcend human shortcomings such as exhaustion or distraction, inter- and intraobserver uncertainty, and the time-consuming and repetitive interpretation of huge data sets [65]. This study’s operating software may be the beginning of the novel technique using 3D-TEE pre-TAVR assessment for the future algorithm of the aortic annular quantification that needs further investigation.

There are some limitations in our study. First, all the included studies were observational studies; Second, statistical heterogeneity(I2) was high for most outcomes; Third, novel software tools using in 3D-TEE for annular measurements were not totally the same commercial software tools; Finally, our study has not reported other parameters of aortic root sizing.

## 5. Conclusions

The present study implies that 3D-TEE using novel software tools for annulus sizing is feasible for MDCT. As the software tool of 3D-TEE does analysis automatically, it is valuable for saving time and reducing operator-dependency which may lead to the future study of artificial intelligence in echocardiography. This application may be used instead of MDCT in some situations, such as avoiding contrast nephrotoxicity, which is particularly necessary for patients who are at high risk of renal failure, or in patients with serious allergies to iodinated contrast, or in cases of arrhythmias that render ECG gating impossible for MDCT acquisition.

## Figures and Tables

**Table 1:** Study characteristics of included studies.

Study/Year	Total No. of Patients	Study Design	Study Period	Hospital/Institution	Country
Thalappillil/2020 [46]	47	Retrospective	1 January 2017 to 1 February 2019	Tufts Medical Center	United States
Maia/2020 [45]	107	Retrospective	December 2015 to January 2017	Centro Hospitalar de Gaia/Espinho	Portugal
Stella/2019 [44]	175	Retrospective	October 2014 to August 2016	San Raffaele University Hospital	Italy
Queiros/2018 [43]	101	Retrospective	August 2014 to September 2017	St. Vinzenz-Hospital (Cologne, Germany)	Germany
Prihadi/2018 [42]	150	Retrospective	NA	The Leiden University Medical Center	The Netherlands
Podlesnikar/2018 [41]	83	Retrospective	July 2015 to March 2017	The Leiden University Medical Center	The Netherlands
Kato/2018 [40]	43	Retrospective	January to October 2016	The Tokyo Bay Urayasu-Ichikawa Medical Center	Japan
Mediratta/2017 [39]	52	Retrospective	NA	University of Chicago Medical Center	United States
Garcia-Martin/2016 [38]	31	Retrospective	March 2012 to March 2014	Ramo’n y Cajal University Hospital	Spain
Khalique/2014 [37]	100	Retrospective	November 2011 to January 2013	Columbia University Medical Center/New York Presbyterian Hospital	United States

NA = Not available.

**Table 2:** Patient characteristics of included studies.

Study/Year	Sex (M/F)	Age (Mean ± SD)	BMI (kg/m^2^) (Mean ± SD)	BSA(m^2^) (Mean ± SD)	Atrial fibrillation (%)	Hypertension (%)	Diabetes Mellitus (%)	AVA (cm^2^) (Mean ± SD)	Mean Transaortic Gradient (mmHg) (Mean ± SD)	LVEF (%) (Mean ± SD)
Thalappillil/2020 [46]	23/24	80.9 ± 7	NA	NA	NA	NA	NA	0.74 ± 0.23	32 ± 12	NA
Maia/2020 [45]	47/60	81 ± 5.9	26.98 ± 4.45	1.74 ± 0.18	37.5	77.5	48.3	NA	49.16 ± 15.23	NA
Stella/2019 [44]	77/98	81.3 ± 6.3	25.2 ± 4.8	NA	30.9%	NA	NA	0.77 ± 0.3	48.4 ± 13.9	56.4 ± 11.3
Queiros/2018 [43]	39/62	83.0 ± 5.1	NA	NA	36.6	91.1	29.7	0.72 ± 0.19	47.4 ± 17.4	54.5 ± 14.6
Prihadi/2018 [42]	74/76	80.7 ± 7.2	26.7 ± 5.5	NA	NA	NA	NA	0.8 ± 0.3	43.5 ± 19.6	50.0 ± 11.8
Podlesnikar/2018 [41]	39/44	81.7 ± 6.7	27.0 ± 4.5	1.84 ± 0.23	NA	NA	NA	0.7 ± 0.2	44 ± 16	57.7 ± 21.5
Kato/2018 [40]	16/27	83.9 ± 4.9	NA	1.45 ± 0.18	NA	81	9	0.58 ± 0.12	47.0 ± 16.8	NA
Mediratta/2017 [39]	28/24	81 ± 8	NA	1.9 ± 0.3	NA	NA	NA	0.8 ± 0.2	40 ± 13	57 ± 16
Garcia-Martin/2016 [38]	10/21	81.6 ± 17	NA	NA	NA	31	7	0.7 ± 0.2	46.3 ± 16	58.2 ± 11
Khalique/2014 [37]	45/55	87.8 ± 8.3	NA	NA	NA	NA	NA	0.67 ± 0.17	NA	NA

NA = Not available, M = Male, F = Female, BMI = Body mass index, BSA = Body surface area, SD = Standard deviation, AVA = Aortic valve area, LVEF = Left ventricular ejection fraction.

**Table 3:** Imaging characteristics of included studies.

Study/Year	3D-TEE		MDCT		Measurement Phase	Software Used for 3D-TEE Annulus Sizing	Sex (M/F)	Time for the AA Analysis (s) (Mean ± SD)
	3D-TEE Technique	Vendor	MDCT Technique	Vendor				
Thalappillil/2020 [46]	Automated	Acuson SC2000 (Siemens)	Manual	128-slice/ 64-slice Dual-Source CT (Siemens)	Systole	eSie Valves AI	23/24	NA
Maia/2020 [45]	Automated	Acuson SC2000 (Siemens)	Manual	64-detector row CT(Siemens)	Systole	eSie Valves	47/60	NA
Stella/2019 [44]	Semiautomated	GE Vivid E9 (GE Healthcare)	Manual	64-slice CT (GE Healthcare)	Systole	4D Auto AVQ	77/98	50 ± 7
Queiros/2018 [43]	Semiautomated	GE Vivid E9/E95 (GE Healthcare)	Manual	Multidetector 64-channel scanner (GE Healthcare)	Systole	Speqle3D	39/62	33.9 ± 9.5
Prihadi/2018 [42]	Automated	iE33 and EPIQ7 (Philips Medical Systems)	Manual	64–detector row /320–detector row CT (Toshiba Medical Systems)	Systole	Aortic Valve Navigator [AVN]	74/76	4.8 ± 1.2 (min)
Podlesnikar/2018 [41]	Automated	GE Vivid E9/E95 (GE-Vingmed)	Manual	320-slice MDCT scanner (Toshiba Medical Systems)	Systole	4D Auto AVQ	39/44	NA
Kato/2018 [40]	Automated	Acuson SC2000 (Siemens)	Manual	320-slice MDCT scanner (Toshiba Medical Systems)	Systole	eSieValves	16/27	30.1 ± 5.79
Mediratta/2017 [39]	Semiautomated	iE33 (Philips Medical Systems)	Manual	256-slice scanner (Philips Medical Systems)	Systole	Mitral Valve Quantification [MVQ]	28/24	NA
Garcia-Martin/2016 [38]	Automated	iE33 (Philips Medical Systems)	Manual	64-slice MDCT (Philips Medical Systems)	Systole	eSieValves	10/21	NA
Khalique/2014 [37]	Semiautomated	iE33 (Philips Medical Systems)	Manual	320-slice MDCT scanner (Toshiba Medical Systems)	Systole	Mitral Valve Quantification [MVQ]	45/55	NA

NA = Not available, 3D-TEE = Three-dimensional transesophageal echocardiography, MDCT = Multidetector computed tomography, AA = Aortic annulus.

**Table 4:** Meta-regression of correlation between 3D-TEE and MDCT annular area sizing.

Variables	No. of Studies	Estimate	Standard Error	95% CI (*p* Value)
Male	6	2.510	1.315	−0.068, 5.088 (*p* = 0.056)
Age	6	0.046	0.036	−0.025, 0.118 (*p* = 0.205)
Left ventricular rejection fraction	4	−0.041	0.031	−0.102, 0.019 (*p* = 0.18)
Aortic valve area	6	0.404	1.420	−2.379, 3.187 (*p* = 0.776)
Mean transaortic gradient	5	−0.044	0.024	−0.090, 0.003 (*p* = 0.065)

3D-TEE = Three-dimensional transesophageal echocardiography, MDCT = Multidetector computed tomography, CI = Confidence interval.

## Data Availability

Not applicable.

## Supplementary Materials

The following are available online at https://www.mdpi.com/article/10.3390/diagnostics11050751/s1, Table S1: PRISMA 2009 Checklist, Table S2: Literature search terms.
