# Supplementary material for: Aortic Annular Sizing Using Novel Software in Three-Dimensional Transesophageal Echocardiography for Transcatheter Aortic Valve Replacement: A Systematic Review and Meta-Analysis
Source: Diagnostics (Basel). 2021 Apr 22;11(5):751. doi: 10.3390/diagnostics11050751 (PMC8145366; doi:10.3390/diagnostics11050751)
Supplement: Supplementary file 1 [file diagnostics-11-00751-s001.zip › Table S2. Literature Search terms- Chanrith Mork.pdf]

SUPPLEMENTARY MATERIALS

Search Terms Used in PubMed, EMBASE, Web of Science, and Cochrane Library (Wiley)

Date: August 30, 2020

Search Terms for PubMed (Title & Abstract): #1AND#2AND#3

- #1: (((((((((((Echocardiography[Title/Abstract]) OR (Two-Dimensional Echocardiography[Title/Abstract])) OR (Three-Dimensional Echocardiography[Title/Abstract])) OR (Transesophageal Echocardiography[Title/Abstract])) OR (Two-Dimensional Transesophageal Echocardiography[Title/Abstract])) OR (Three-Dimensional Transesophageal Echocardiography[Title/Abstract])) OR (2D-TEE[Title/Abstract])) OR (3D-TEE[Title/Abstract])) OR (2D-TOE[Title/Abstract])) OR (3D-TOE[Title/Abstract])) OR (((Novel software[Title/Abstract]) OR (automated software[Title/Abstract])) OR (semi-automated software[Title/Abstract])))

- #2: (((((((((((Multidetector Computed Tomography[Title/Abstract]) OR (Multislice Computed Tomography[Title/Abstract])) OR (Multidetector-Row Computed Tomography[Title/Abstract])) OR (Spiral Computed Tomography[Title/Abstract])) OR (X-Ray Computed Tomography[Title/Abstract])) OR (Spiral Computer-Assisted Tomography[Title/Abstract])) OR (Helical Computed Tomography[Title/Abstract])) OR (MDCT[Title/Abstract])) OR (MSCT[Title/Abstract])) OR (Spiral CT Scans[Title/Abstract])) OR (Helical CT[Title/Abstract])) OR (CT Scans[Title/Abstract])))

- #3: (((((Transcatheter Aortic Valve Implantation [Title/Abstract]) OR (Transcatheter Aortic Valve Replacement [Title/Abstract])) OR (Aortic valve stenosis [Title/Abstract])) OR (TAVI[Title/Abstract])) OR (TAVR[Title/Abstract]))

Search Terms for Embase (Title & Abstract): #1AND#2AND#3

- #1: Echocardiography OR Two-Dimensional Echocardiography OR Three-Dimensional Echocardiography OR Transesophageal Echocardiography OR Automated OR Semi-automated OR Novel software OR New Software OR 2D-TEE OR 3D-TEE OR 2D-TOE OR 3D-TOE

- #2: Multidetector Computed Tomography OR Multislice Computed Tomography OR Multidetector-Row Computed Tomography OR Spiral Computed Tomography OR Helical Computed Tomography OR CT scans OR MDCT OR MSCT

- #3: Transcatheter aortic valve implantation OR Transcatheter aortic valve replacement OR Heart Valve Prosthesis OR Aortic Valve Stenosis OR TAVI OR TAVR

Search Terms for Web of Science (Topic= Title & Abstract & Source) : #1AND#2AND#3

- #1: TS = (Echocardiography OR Two-Dimensional Echocardiography OR Three-Dimensional Echocardiography OR Transesophageal Echocardiography OR Automated OR Semi-automated OR Novel software OR New Software OR 2D-TEE OR 3D-TEE OR 2D-TOE OR 3D-TOE)

- #2: TS= (Multidetector Computed Tomography OR Multislice Computed Tomography OR Multidetector-Row Computed Tomography OR Spiral Computed Tomography OR Helical Computed Tomography OR CT scans OR MDCT OR MSCT)

- #3: TS=(Transcatheter aortic valve implantation OR Transcatheter aortic valve replacement OR Heart Valve Prosthesis OR Aortic Valve Stenosis OR TAVI OR TAVR)

Search Terms for Cochrane Library (Wiley) [Journals]: **#1AND#2AND#3**

- #1: Transcatheter aortic valve implantation OR Transcatheter aortic valve replacement OR Aortic valve Stenosis OR TAVI OR TAVR
- #2: Echocardiography OR Two-Dimensional Echocardiography OR Three-Dimensional Echocardiography OR Transesophageal Echocardiography OR 2D-TEE OR 3D-TEE OR 2D-TOE OR 3D-TOE OR Automated OR Semiautomated OR New software OR Novel software
- #3: Multidetector Computed Tomography OR Multislice Computed Tomography OR Multidetector-Row Computed Tomography OR Spiral Computed Tomography OR Helical Computed Tomography OR CT scans OR MDCT OR MSCT
